# Supplementary material for: Are carbon emissions trading and green financial instruments synergistic? -Comprehensive quantitative research based on content analysis
Source: PLoS One. 2024 Mar 7;19(3):e0298601. doi: 10.1371/journal.pone.0298601 (PMC10919601; doi:10.1371/journal.pone.0298601)
Supplement: S1 Data — (ZIP) [file pone.0298601.s001.zip › Data/地级市Z_F工作报告.zip!/╣π╢1⁄2/╔╟═╖╩╨/└┤╫╘╣1⁄2╓┌║┼í╛┬φ┐╦╩2╛▌═°í┐_2017.docx]

各位代表:

现在,我代表汕头市人民政府,向大会报告工作,请予审议,并请各位政协委员和其他列席人员提出意见。

一、工作回顾

过去五年,市政府在省委、省政府和市委的正确领导下,以党的十八大和省第十一次党代会精神为指导,学习贯彻习近平总书记系列重要讲话精神和治国理政新理念新思想新战略,紧紧围绕省委、省政府“三个定位、两个率先”目标,深入落实省创新驱动发展和粤东西北振兴发展战略,保持经济社会平稳较快发展,顺利完成市第十三届人代会提出的主要目标任务。五年间,地区生产总值年均增长9.1%,一般公共预算收入年均增长10.2%,固定资产投资额年均增长30.4%,社会消费品零售总额年均增长12.5%,实际吸收外资年均增长13.6%,对外投资额年均增长66.6%。

特别是2016年,我们扎实推进供给侧结构性改革,以创建全国文明城市为总抓手,大力开展城市环境治理,搭建发展平台、提升对外开放水平,促使经济发展环境迅速改善,人气和资源迅速集聚,呈现发展提速、质量提升、排位提前的良好态势,实现“十三五”良好开局。预计2016年全市实现生产总值2055亿元,增长8.7%,增速居全省前列;一般公共预算收入137.1亿元,增长6.2%;固定资产投资额1574亿元,增长23.5%;社会消费品零售总额1511亿元,增长12%;外贸进出口总额576亿元,规模列粤东西北地区第一位。

(一)狠抓供给侧结构性改革,体制机制激发新活力。出台“三去一降一补”行动计划,出清重组国有“僵尸企业”155家;超额完成房地产去库存任务;化解地方金融风险,处置涉众金融案件7宗、涉案金额1.25亿元;实施7方面35项措施为企业降成本60亿元;完成农村配电网改造升级项目624个,新建公众移动通信基站超过7000座,光纤入户率超过60%。创新投融资机制,设立规模100亿元的城市发展基金和91个PPP项目,获国家专项建设基金支持项目8个、投资规模63.6亿元,获国开行、农发行融资批复超过47亿元。深化财税体制改革,全面推开“营改增”试点。实施“五证合一、一照一码”等商事登记制度改革,新登记企业7391户、注册资本382.87亿元,分别增长29.3%、110.6%。深化关检作业无纸化、通关一体化改革,大通关环境进一步优化。

(二)狠抓重大项目重大平台建设,城市发展开拓新空间。全面推进港口、铁路、高速公路等基础设施建设。广澳港区2个10万吨级集装箱泊位完成投资量25%,8.3公里防波堤工程完成6公里,航道二期工程开工建设。广梅汕铁路增建二线及厦深铁路联络线完成工程量63%,汕头火车站综合客运枢纽动工,疏港铁路启动工程可行性研究报告编制,城市轨道交通完成线网规划编制、启动建设规划编制及试验段建设。潮惠高速公路汕头段建成通车,揭惠高速公路汕头段完成投资量60%,汕湛高速公路汕头段、潮汕环线高速公路一期开工建设。海湾隧道主体工程开工,泰山路北延、磊口大桥扩建工程建成通车,国省道完成改造122公里,新建农村公路150公里。全面推进“三大平台”建设。华侨经济文化合作试验区加快建设,东海岸新城累计完成投资130亿元,16.4公里滨海大道全线贯通,新津片区部分围填海项目竣工验收,国际科创金融城、科创园、国际潮商总部大厦动工建设。汕头高新区即将升格为国家级高新区;广东以色列理工学院正式设立,一期校区主体结构全面封顶;中以(汕头)科技创新合作区启动基础设施建设。临港经济区启动总体规划编制,汕头保税物流中心(B型)启动建设。

(三)狠抓新技术新产业新业态,实体经济释放新动能。高新技术企业培育成效显著,净增175家,总量增长117%。新增省级工程技术研究中心10家、省级新型研发机构4家。加强知识产权保护与应用,发明专利申请量和授权量分别增长34.9%和12%,中国汕头(玩具)知识产权快速维权中心揭牌。获批创建“全国质量强市示范城市”和“全国服装(内衣家居服)产业知名品牌示范区”,国家玩具质量监督检验中心大楼封顶。出台实施促进民营经济大发展大提升的50条措施、支持上市企业做大做强的15条措施、“1+10”人才政策等,设立首期20亿元的股权投资基金、规模1亿元的中小微企业信贷风险补偿资金、规模1.3亿元的政策性担保资金,开通政企通APP平台,全力破解制约企业发展的系列难题。培育战略性新兴产业取得突破,比亚迪跨座式单轨、美中时空通用航空等项目开工建设,与三峡新能源公司开展海上风电产业合作。金融加快发展,入选国家产融合作试点城市,获批设立全国首个以华侨为主题的广东华侨金融资产交易中心,新增金融主体4家,金融机构总数达90家,A股上市企业、“新三板”挂牌企业、“华侨板”挂牌企业分别达27家、47家、432家。“互联网+”新业态蓬勃发展,电子商务交易量增长30%,淘宝镇、淘宝村数量分别增至11个、58个,列全省第一、第二位,连续两年进入全国“电商百佳城市”30强。特色农业、海洋渔业和休闲旅游业加快发展,县级以上农业龙头企业和农民专业合作社分别达85家和983家;南澳县获评首批国家级海洋生态文明建设示范区,“蓝色海湾”整治项目获中央扶持资金3亿元;潮阳区海门镇获评全国特色景观旅游名镇,潮南区东华村获评中国最美休闲乡村。

(四)狠抓城市建设管理,城乡面貌呈现新气象。开展全国文明城市创建活动,实施城市环境“九大提升行动”,一批“老大难”问题得到解决。拆除违章建筑物27.8万处,完成新兴路、中山西路、长平路等17.97万平方米破损道路,以及嵩山路、衡山路、金新路等19.62万平方米步道改造,打通庐山北路、金环南路、金新北路等一批困扰多年的“断头路”,海滨长廊13个码头开口连接贯通。时代广场、西堤公园、中山公园、北郊公园等10个公园,新大华、新华坞等13个农贸市场完成改造升级。小公园开埠区全面启动保护性改造,基本完成小公园中山纪念亭原貌修复、西堤路沿路建筑物景观整治和外立面修缮,保育修复桂园、广东东江各属行政委员公署旧址等10个历史文物,改造提升首批15家知名“老字号”特色美食店。加强生态环境治理,组建环保警察,出台奖励公众举报环境违法行为办法及系列追责措施,严厉打击各类环境违法行为。贵屿环境综合整治通过省验收,基本建成占地950亩的循环经济产业园区,基本实现规划、建设、运营、治污、监管“五统一”管理。练江流域综合整治艰难破题,关停取缔印染企业210家、造纸企业20家,关闭流域内零散养殖场(户)3621家;已建和在建污水处理厂10座、管网83.6公里;潮南区纺织印染园污水处理厂首期主体工程基本完成,练江水质指标明显改善。连续三年开展“百河千沟万渠大整治”行动,整治河沟717宗。推进森林进城、森林围城,建设生态景观林带36公里、碳汇林1.41万亩、森林公园和湿地公园7个,绿化美化村庄42个。

(五)狠抓民生热点问题解决,社会事业取得新成效。民生支出占公共财政支出超过70%。实施精准扶贫、精准脱贫攻坚工程,投入3.55亿元,完成3.3万相对贫困人口脱贫。新增城镇就业5.3万人、农村劳动力转移就业4.1万人。实现国家义务教育发展基本均衡县(区)、省教育强县(区)全覆盖,获广东省教育强市称号。普通高考总上线率、中职学校毕业生就业率分别连续五年超过90%、96%。全面启动“卫生强市”建设,新改扩建8个区县级医院、11个乡镇卫生院、7个社区卫生服务中心,新增三甲医院1家、三级医院6家、省级重点医学专科16个、床位3539张,粤东医疗中心地位进一步增强。社会保障制度基本实现全覆盖,城乡居民医保人均补助标准、企业退休人员月人均养老金分别提高到420元、1962元。优先发展公共交通,新增投放公交车250辆。深入开展“平安汕头”建设,大力开展“三类重点地区”整治,全市刑事、治安警情、交通事故死亡人数分别下降12.4%、13.6%、1.9%。狠抓安全生产事故隐患排查治理,全市未发生较大以上生产安全事故。完善粮食安全政府责任制,提升粮食物流、仓储设施功能。我市法治民政经验得到全国推广。第六次蝉联“全国双拥模范城”。获评全省人民防空先进城市。国防动员、体育、计划生育、殡改、民族、宗教、外事、侨务、港澳事务、对台、工会、妇女儿童、共青团、应急管理、信访、气象、地震、档案、新闻出版、科协、社科、地方志等各项工作取得新进展。2016年确定的十件民生实事完成年度任务。

(六)狠抓政府自身建设,行政效能实现新提升。自觉接受人大、政协监督,办理人大议案、建议86件和政协提案154件,提请市人大常委会审议法规草案3件、制定和修改政府规章17件。出台法治政府建设“十三五”规划。法律援助规范化发展,设立法律援助志愿律师库和8个法律援助便民服务窗口。简政放权优化服务改革深入推进,出台政府部门权责清单管理办法,取消、调整74项市级行政审批事项和50项中介服务事项,清理市政府规章、规范性文件588件。完成行政审批标准化建设,公布市级行政许可和公共服务事项目录,实现审批办事时限提速50%以上。完成“汕头政府在线”建设,实现后台审批、前台服务全程电子化。深入推进“一门式一网式”政府服务模式改革,分类整合服务窗口,市网上办事大厅进驻单位45个、进驻事项829项,行政审批事项网上全流程办理率达69%。全面推进财政预决算、“三公”经费和重点领域信息政务公开。扎实开展“两学一做”学习教育活动,严格落实中央“八项规定”,坚决纠正“四风”问题。深入开展重点工作“百日攻坚”行动,推广“三定一督办”和“三集中”工作方法,坚持用结果说话,干部队伍的执行力和落实力明显增强,行政效能明显提升,政府作风明显改进。

各位代表!过去五年,我们所取得的成绩和进步,是省委、省政府和市委正确领导的结果,是全市人民同心协力、共同奋斗的结果,是海内外乡亲大力支持、积极参与的结果。在此,我代表市人民政府,向全市广大工人、农民、知识分子、干部职工,向给予我们支持的人大代表、政协委员、各民主党派、工商联、社会各界人士和海内外乡亲,向中直和省直驻汕单位,向驻汕解放军、武警官兵,向所有关心和支持汕头发展的港澳台同胞、海外侨胞和国际友人,表示崇高的敬意和衷心的感谢!

但我们也清醒地认识到,与人民群众、海内外潮人的期盼相比,我市经济社会发展还存在很多亟待解决的问题:一是企业自主创新能力不强,产业层次偏低,新经济新产业尚未形成规模,传统产业转型升级任务重,实体经济困难较多。二是人才总量与结构难以适应新时期发展的需要,创新型人才、高技能人才的引进培养力度需进一步加大。三是民生保障任务重,医疗、教育、公共交通等公共服务均等化程度不高,部分群众生活还比较困难。四是城乡环境污染还比较严重,生态治理任务紧迫而艰难。五是城市管理精细化水平不高,市容市貌综合整治任重道远,特别是中心城区交通拥堵、交通秩序混乱等问题突出。六是政府职能转变还不到位,审批难、办事难依然是企业和群众反映强烈的问题。对此,我们必须采取更加有力的措施,切实加以解决。

二、今后五年目标任务

各位代表,前不久,我们胜利召开中共汕头市第十一次代表大会,作出了加快推动汕头全面振兴发展的重大战略部署。按照市第十一次党代会的部署,全市上下将围绕“五位一体”总体布局和“四个全面”战略布局,牢固树立和贯彻落实新发展理念,积极参与“一带一路”建设,以全面振兴为主题,以创建全国文明城市为抓手,以创新驱动为核心,以产业高端发展为重点,着力提高经济发展的质量和效益,加快建设华侨试验区、国家级高新区和临港经济区,构筑大汕头湾区城市格局,提升粤东中心城市地位,努力实现经济社会大变化大发展。

今后五年,我市经济社会发展的主要奋斗目标是:到2021年,全市生产总值达到3400亿元以上,年均增长9%以上。为实现这个目标,要突出做到“五个坚持”:

坚持民生为本。落实以人民为中心的发展理念,顺应民心、尊重民意,感知群众冷暖,回应群众关切,扎实推进基本公共服务均等化,加快形成全覆盖、可持续的社会保障制度和更加健全完善的基本公共服务体系,努力提高人民群众生活水平,确保到2018年城乡居民人均收入比2010年翻一番。

坚持环境优先。持之以恒推进创文强管,促进城乡环境显著改善,2017年取得全国文明城市提名资格,2020年获得全国文明城市称号。强力治理环境污染,提升空气质量,着力破解水污染治理难题,2021年城镇生活垃圾无害化处理率、生活污水处理率分别达到95%、98%,争创国家森林城市、国家生态园林城市、国家节水型城市、国家绿色发展示范区。

坚持创新驱动。扎实推进供给侧结构性改革,深入实施创新驱动发展战略,完善促进创新政策体系,大力引进培养创新型人才,建设创新型特区。改造提升传统产业,培育一批战略性新兴产业和先进制造业产业集群,着力振兴实体经济。全面提升产品、工程、服务和环境质量,创建“全国质量强市示范城市”。2021年,研究与试验发展经费支出占地区生产总值比重达到2.8%以上,每万人拥有发明专利2.45件以上,高技术及先进制造业增加值占规模以上工业比重达到30%,公民具备科学素质比例达到10.5%以上。

坚持投资拉动。以补齐基础设施短板为重点,突出抓好高速公路、铁路、重大水利工程、新一代信息基础设施及能源、环保基础设施建设。进一步加大工业投资和技术改造投资力度,促进工业经济转型升级。进一步激活民间投资,引导社会资金更多投向实体经济和基础设施建设薄弱环节,更好发挥民间投资的主力军作用。未来五年累计完成固定资产投资额超万亿元。

坚持开放带动。加快推进“一带一路”重要门户城市建设,争取华侨试验区纳入国家自由贸易试验区范围,建立与国际通行规则相衔接的体制机制,促进投资贸易便利化,构筑全方位、多层次开放合作新格局。发挥海内外潮人潮商优势,创新引侨聚侨机制,以侨引智、以侨引外,建设国家通侨联侨重要枢纽。

三、2017年工作安排

总体要求是:全面贯彻党的十八届六中全会精神,深入贯彻习近平总书记系列重要讲话精神,按照中央经济工作会议、省委十一届八次全会、省委经济工作会议精神和市第十一次党代会、市委十一届二次全会的部署要求,坚持稳中求进工作总基调,坚持以提高发展质量和效益为中心,坚持以推进供给侧结构性改革为主线,深化创新驱动,振兴实体经济,全面做好稳增长、促改革、调结构、惠民生、防风险各项工作,促进经济平稳较快增长和社会和谐稳定。

今年经济社会发展的主要预期目标是:地区生产总值增长9%以上,固定资产投资额增长25%以上,社会消费品零售总额增长12%以上,外贸进出口总额增长6%以上,地方一般公共预算收入可比增长6%以上,居民消费价格涨幅控制在3%以内,居民人均收入增长9.5%以上,城镇登记失业率控制在3%以内,节能减排降碳约束性指标完成省下达年度计划。重点抓好以下六方面工作:

(一)加快“三大平台”建设,着力打造新型经济增长极

加快建设华侨试验区。加大改革创新力度,积极争取国家和省政策落地,争取纳入国家自由贸易试验区范围。推进“粤港澳服务贸易自由化省级示范基地”建设,加快国际科创金融城、科创园等现代产业项目建设,致力打造区域科创中心。做强做大华融华侨资产管理平台,启动运营华侨金融资产交易中心,推动设立国家级华侨产业母基金、华侨银行、华侨人寿保险等一批金融服务平台和机构,构建具有侨特色的金融政策试点区。创办华侨创业创新服务中心和国家、省双创基地以及省人才创新示范基地,争取“侨商智库研究院”等项目落户。

加快建设国家级高新区。推进“政区合一”,加快中以(汕头)科技创新合作区建设,高起点规划建设5.6平方公里起步区,动工建设金凤西路二期等基础设施。推动设立中以航空产业基金和中以技术转移中心,重点发展生物制药、航空产品制造、新材料、文化创意等产业,着力打造创新创业新引擎、产城融合示范区。

加快建设临港经济区。坚持港航并重、港区联动、港城融合。广澳港区2个10万吨级集装箱泊位、防波堤、航道二期工程同步建成。引进大型码头运营商、航运龙头企业,参与港区综合运营开发,完善港口配套服务和集疏运体系。以广澳港区、海门港区为核心,加快规划建设临港物流中心和临港工业园,推进保税物流中心建设,大力发展港口航运、装备制造等现代临港产业。

(二)强化创新引领,着力发展实体经济

深入推进“三去一降一补”。淘汰落后产能,积极稳妥出清“僵尸企业”。落实房地产调控政策,逐步消化房地产库存。加快建立购租并举的住房制度,推进专业化住房租赁平台试点工作。积极防范地方债务风险,加强金融监管,坚决守住不发生区域性系统性金融风险的底线。加大降费、降低要素成本工作力度,促进企业降本增效。补齐软硬基础设施短板,增加公共产品和公共服务供给。

强化科技创新。加大创新资金扶持力度,设立中小型科技企业创新基金、科技创新研发风险基金、新型研发机构建设专项资金。大力培育高新技术企业,新增200家以上。健全研发体系,加快华中科技大学汕头新材料研究院、重庆交通大学金东海研究院、广东石油化工学院美联化工研究院等一批科研中心建设,新增新型研发机构3家以上。加快科技平台和创新平台建设,鼓励发展工程技术研究中心、工程实验室、企业科技中心等科技平台,推动粤东科创中心、中海信创新产业城、汕头工业设计创意中心、“一带一路”互联网+创客孵化基地、纺织产业服务平台等建设,新增科技企业孵化器5家以上。实施知识产权优势企业培育工程,支持发明专利申请和PCT专利申请。推进新一轮工业企业技术改造,开展汕头市智能制造2015—2025行动,加快“装备工业两化深度融合暨智能制造试点”和“广东省智能制造示范基地”建设,推动传统产业开展机器人应用,培育智能制造示范企业10家以上。深入实施各类人才计划,制定更具吸引力的人才政策,加大创新型、技能型人才引进培育力度。高起点、高标准建设市级高层次人才创新、创造、创业基地。大力引进海外高端科技人才和项目,增设海内外人才工作联络站15个。

壮大现代产业。培育发展轨道交通、通用航空、新能源、大数据等战略性新兴产业,争取形成新的支柱产业。推动比亚迪跨座式单轨项目投产,吸引上下游关联企业集聚。美中时空通用航空小型飞机项目投产,与以色列航空工业公司合作项目落地。推进中节能贵屿园中园项目建设,加快海上风电电机产业链、濠江粤电LNG清洁能源、华南石墨烯新材料创新产业园等项目落地。推进建设广东联通粤东云计算大数据中心等项目,打造粤东云计算、大数据交易中心。加快发展金融服务业,实施“引金入汕”工程,大力引进境内外银行、证券、期货、保险、基金等金融机构。推进国家产融合作试点城市建设,创新产业园区提质增效融资模式、科技创新平台产融合作模式,帮助中小微企业解决融资难问题,加大金融对实体经济的支持力度。鼓励企业改制上市,争取新增境内外上市企业和“新三板”挂牌企业15家。大力发展商贸物流业,推动华润万象城、潮阳内衣商贸城、潮南巴比伦商贸城等大型商贸项目,京东粤东电商物流基地、苏宁易购粤东运营中心等现代物流园区建设,争取宝奥城列入国家市场采购贸易试点。做大做强旅游业,推进内海湾旅游圈、海洋旅游、侨文化、古村落、红色旅游等开发。高水平规划建设南澳岛,全力推动南澳岛创建国家5A级旅游景区。支持濠江区、南澳县与中国青旅实业集团等大型企业合作开发旅游资源。鼓励发展影视传媒、动漫游戏、产品设计等文化创意和设计产业。

创建质量强市。支持各区(县)创建“广东省质量强市(县、区)示范区”,加快澄海国家级出口玩具质量安全示范区和玩具质量追溯体系试点建设。实施名牌带动战略,培育名牌产品和商标品牌,启动创建“全国动漫产业(玩具)知名品牌示范区”。支持企事业单位主导或参与各级标准的制修订。加强公共技术服务平台建设,成立质量发展科学研究中心,加快国家玩具质检中心、国家洗涤护肤用品质检中心和省轻工机械产业计量测试中心建设,争取多功能包装机械、口腔清洁用品等省级检验检测站获批筹建,构建与产业发展相配套的公共检测技术服务体系。

培育新兴业态。实施小微企业创新创业基地城市示范三年行动计划,积极发展众创、众包、众扶、众筹等新业态、新模式。实施“互联网+”行动计划,积极与腾讯、京东、九次方等企业开展多领域合作。加快发展电子商务,培育电商龙头企业,推动淘宝、苏宁等电商平台“汕头馆”建设。推进跨境电商公共服务平台建设,培育、引进大型外贸综合服务企业,力促外贸企稳向好。培育发展智能家电、智能玩具等物联网新业态。

优化产业载体。加快产业园区扩能增效,推进金平现代产业集聚区、龙湖东部新兴产业园、中国锆城、南山湾科技产业园、潮阳贵屿产业园建设,加强基础设施配套,积极创建省级产业园区。筹建金平医药孵化园,打造大健康产业基地。支持发展工业地产,开展楼宇产业园、标准厂房建设试点。扶持发展特色小镇,推进专业镇协同发展,加快建设一批特色产业协同创新中心。

发展特色农业。深入推进农业供给侧结构性改革,加强农产品标准化生产,加大农业科研投入,增加绿色优质农产品供给。培育发展农业龙头企业、农民专业合作社、家庭农场等新型农业经营主体,力争新增市级以上农业龙头企业3家、农民合作社及家庭农场30家。加快发展互联网+现代农业,建设南澳北斗海通渔业电商平台等农村农业电商,推进省级现代农业示范区建设。推进粮食市场化改革,完善粮食安全保障体系。深化供销合作社综合改革,深入推进“新网工程”建设。扶持发展休闲农业与乡村旅游业,推进特色旅游名镇、名村建设。发展现代渔港经济,加快濠江国家一级渔港、南澳后江现代渔港建设。推进“蓝色海湾”整治项目、海洋牧场建设。

(三)加快城市扩容提质,着力提升全市域一体化水平

加快新城新区规划建设。开展大汕头湾区规划研究,加强陆海统筹,增强区域中心城市集聚辐射功能。东海岸新城整体完成用海验收、纳入允许建设区,新津片区8条市政道路和莱芜大桥竣工验收,国家示范性高中和国际学校项目完成主体工程建设,启动建设22.64公里综合管廊和新溪片区市政道路。珠港新城加快国际潮商、超声研究所、联泰等总部项目建设,泰山路南延、中山东路东延、珠港路等市政设施动工。粤东物流新城加快北片区土地一级开发、跨梅溪河大桥等项目建设,推动一批高端物流总部企业落户。濠江新城建成潮汕历史文化博览中心、岸线景观工程(一期)、南滨路东段、南滨南路中段,启动建设濠州路、山体雕塑公园、红树林湿地公园。科学谋划榕江经济走廊、牛田洋片区、新津河两岸开发建设。加强城市天际线规划控制,提升“一湾两岸”岸线景观。

加快老城区修缮改造。以“三环三线”为重点,全面加快小公园历史文化街区保育活化,重点推进永平路等老街区注入新业态,打造一批百年老街品牌。修缮老妈宫戏台、红色交通站、彬园,改造第二批特色美食店。规划建设开埠邮局陈列馆、对外贸易陈列馆、教育博物馆等,打造开埠文化专题博物馆群。启动乌桥岛棚户区改造,推进搬迁安置工作。加快旧村庄改造,加强农村土地管理,鼓励建设农民公寓。

加快完善综合交通体系。围绕构筑大汕头湾区,加快铁路、高速公路、干线公路建设,打造中心城区便捷内环和区域快速外环交通路网。铁路方面,广梅汕铁路增建二线及厦深铁路联络线通车,建成汕头火车站综合客运枢纽首期工程、城市轨道1号线部分基建工程及相应试验线,启动建设疏港铁路,积极谋划沿海高铁。高速公路方面,揭惠高速汕头段建成通车,加快建设汕湛高速汕头段,推进潮汕环线高速一期全线征拆和控制性工程。干线公路方面,加快建设中心城区与各区县的干线通道,金凤路高架桥、省道335线澄海段建成通车,推进建设海湾隧道主体工程、虎头山隧道、省道336线南澳环岛公路,启动建设牛田洋快速通道、国道324线礐石大桥至红旗岭段、国道539线澄莱段、省道232线护堤路段。新建农村公路150公里。

加快智慧城市建设。推进数字化城市管理建设,实现中心城区范围内市政、园林、环卫、城管执法、道路停车信息化管理全覆盖。统筹建设医疗、卫生、教育等民生领域的智慧应用项目。加快电动汽车充电桩规划建设。持续推进“宽带中国”示范城市创建,重点推进光纤网络、移动通信基站、公共无线局域网络建设及宽带应用。

(四)坚持以创文强管为抓手,着力改善城乡人居环境

突出“治水”。加强水环境治理,加大饮用水源水质保护力度,强化韩江流域联防联治,推进梅溪河、鸥汀片区水域治理。全面实施“河长制”,以最坚决的态度、最严格的制度、最有力的举措,以断源截污、清淤清障、生态修复为重点,推进练江流域综合整治。潮南区纺织印染园争取实现首批印染企业入园生产,潮南区生活垃圾焚烧发电厂点火试运行,潮阳区生活垃圾填埋场无害化升级改造工程完工、垃圾焚烧发电厂启动建设,和平、关埠、铜盂污水处理厂建成运营,潮阳、贵屿、谷饶、峡山、两英污水处理厂提标改造工程全面完成。按照“先支流、后干流”综合整治模式,先行开展潮阳北港河、潮南峡山大溪等重点支流集中治理。加快龙湖沟、新河沟、星湖公园、港区排洪沟等13宗黑臭水体整治,继续开展“百河千沟万渠”大整治。完善水上环卫管理机制,加强水域市容管理。加快水利基础设施建设,推进粤东灌区、村村通自来水、潮阳潮南引韩工程,建成三屿围海堤、海门堤围等工程。

铁腕“治污”。以落实中央环保督察交办案件整改工作为抓手,着力解决一批环保突出问题。巩固提升贵屿环境污染整治成果,改造提升生产工艺,提高贵屿循环经济产业园区运营管理水平。强化机动车污染防治,基本完成黄标车淘汰任务。强化工业污染源大气污染治理,淘汰改造禁燃区高污染燃料锅炉。实施土壤污染防治行动计划,推进贵屿土壤修复工程。

强力“治脏”。大力实施城乡清洁工程,突出整治背街小巷、农贸市场、城乡结合部的环境卫生。推广“24小时巡查监管+半小时快速处置”城市管理模式,全面推行环卫作业市场化改革。扎实推进“美丽乡村、幸福村居”创建活动,加快省级新农村示范片建设,实施“三清三拆三美化”村庄整治行动,推动创文强管工作向农村基层延伸,实现全市乡村环境大转变大提升。深入开展新一轮绿化大行动,建设碳汇林1.56万亩、沿海防护林1.1万亩。

综合“治乱”。制定实施中心城区交通改善三年行动计划,以建设智能交通、完善道路网络、倡导绿色出行为重点,着力缓解主城区交通拥堵。启动泰山路、黄河路、金凤路快速化改造工作,加快推进黄河路东延、金砂路西延、中山路西延和天山北路、长江路、汕樟路等一批市政道路的改造建设。鼓励民间资本参与建设停车场,加强停车规范管理,破解停车难、停车乱问题。坚持疏堵结合,开展电动自行车登记试点等工作,综合治理摩托车、电动自行车乱象。规划建设一批人行过街设施,缓解道路人车冲突及行人过街难。大力发展公共交通,推进快速公交首期工程建设,新增投放新能源公交车400辆以上,增加定制公交线路;加快城乡公交一体化,实现跨区(县)公交线路达25条以上;中心城区建成区公交站点500米服务半径覆盖率达到98%,群众搭乘公交更加便捷。稳妥推进出租汽车改革,规范网约出租汽车经营秩序。

(五)加快补齐民生短板,着力提高人民生活水平和质量

实施精准扶贫攻坚工程。坚持规划到户、责任到人,完善贫困人口的教育、医疗、住房“三保障”等政策,扎实推进产业、就业、社会保障、基础设施建设等扶贫攻坚八大工程,精准实施相对贫困村、贫困户三年帮扶规划,促进贫困人口增收,确保实现4万人脱贫。

实现更加充分更高质量就业。健全政府激励创业、社会支持创业、劳动者勇于创业新机制,推广“互联网+创业孵化基地”新模式,推进保税区“省市共建跨境电子商务创业孵化基地”建设。构筑集产业集聚、市场培育、企业孵化、服务拓展等功能于一体的汕头人力资源服务产业园。新增城镇就业5万人、农村劳动力转移就业3万人。

全面推动教育现代化建设。深入推进教育现代化先进县(区)、先进市创建工作,全面实施“强师工程”及“平安校园工程”,加快推进教育信息化,力争4个区(县)申报教育现代化先进县(区)。高标准发展15年基础教育,扶持一批普惠性规范化民办幼儿园,增加优质幼儿园学位;全面实施初中学校品质提升三年行动计划,打造一批优质品牌公立初中学校;加快普通高中多样化内涵发展,推动一批公办高中学校达到省一级以上的办学标准。改革发展中职教育,整合优化中职学校,创建广东省现代职业教育综合改革示范市。加快发展高等教育,支持汕头大学建设国内先进、国际知名的高水平大学,争取汕头职业技术学院建成省级示范性高职院校,办好汕头技师学院;完成广东以色列理工学院一期校区建设,实现招生办学;整合市卫生学校与金平职业技术学校资源,建设市卫生健康学院,规划建设汕头国际奥林匹克体育学院。发展特殊教育、继续教育和社区教育,推动市电大业大职大转型为开放大学。加强师德师风建设,营造尊师重教氛围。

深入推进“卫生强市”建设。全面实施城市公立医院改革,推行分级诊疗制度,引导更多患者到基层医疗机构就诊。提升医疗服务能力,新改扩建汕大肿瘤医院、汕头国际眼科中心、市中医院、民生医院等12家三级医院,新增2家三甲医院,完成县级医院基本设备配置和全部乡镇卫生院标准化建设,确保基层医疗卫生机构诊疗量占全市的60%以上。加快医疗卫生信息化建设,实现全市医疗卫生机构资源共享和信息互通。加大力度支持社会办医,社会办医服务总量达到30%。实施三级医院帮扶全市30家乡镇卫生院三年行动计划,提升基层医疗卫生水平。完善公共卫生服务体系,推动区县疾控机构达标建设,有效预防控制疾病。深化人口计生综合改革,有效管控“全面两孩”政策后的人口风险。加强妇幼保健服务能力建设,启动异地建设市妇幼保健医院,筹建市中心医院儿童专科医院,加快建设汕大附一院、附二院儿科中心,着力解决儿童看病难问题。加强医德医风建设,构建和谐医患关系。

加快文化体育事业发展。新改扩建图书馆、档案馆、博物馆等公共文化设施,全面完成农家书屋改造升级,推进基层综合文化服务中心、社区文化广场、农村公益电影固定放映点建设。加强城市文化形象推介,打造城市文化品牌。落实全民健身国家战略,开展社区体育设施全覆盖行动,新增社区健身场地195个、体育设施面积25万平方米。继续办好汕头国际马拉松、“潮人杯”国际帆船等赛事。

不断完善社会保障体系。提高底线民生保障水平,城乡低保月人均补差水平提高到不低于457元和206元,农村五保供养标准不低于上年度当地农村居民人均可支配收入的70%。加强保障房建设,新开工棚户区改造2210套,新增发放租赁住房补贴260户。推动养老事业发展,继续实施普惠型高龄津贴制度和银龄安康行动,探索医养结合养老模式,加快规划建设养老服务中心和临终关怀服务机构,鼓励民办养老机构发展,各类养老床位达1.8万张。完善社会救助体系,加大对孤儿、残疾人、精神病患者、流浪人员等特殊群体救助力度。加大社保扩面征缴工作力度,确保参保人数和缴费人数同步增长,改善养老保险基金收支状况。弘扬公益文化,发展慈善事业。

提升社会治理水平。深化“平安汕头”建设,完善立体化治安防控体系,严打“两抢一盗”、涉毒、涉税、涉假、涉污等突出违法犯罪行为,不断提高人民群众安全感。全面加强公安工作,坚持素质强警、从严治警、从优待警,加快正规化警队建设。抓好综治信访维稳,依法规范信访秩序,完善调解、仲裁、诉讼等社会矛盾预防和化解机制。加强农村“三资”管理,促进农村基层社会稳定。完善“党政同责、一岗双责、齐抓共管、失职追责”责任体系,创新社会消防网格管理,深入开展事故隐患排查治理和“双盲”演练,保持全市安全生产形势稳定好转。强化应急管理,提高突发事件防范处置和防灾救灾减灾能力。加强食品药品安全监管,严厉打击制假售假违法行为,确保人民群众饮食用药安全。支持和规范社会组织承接政府购买服务。大力发展志愿服务事业。支持国防和军队改革,做好国防动员、“双拥”优抚工作,推进军民融合深度发展。做好民族、宗教、人防、殡改等各项工作。

继续办好十件民生实事。计划投入资金17.7亿元,集中力量办好十件民生实事。一是落实底线民生保障,提高生活补助标准。二是加大教育保障投入,促进教育均衡发展。三是落实居民医保补助,改善城乡医疗卫生服务。四是推进农村创文,改善农村生产生活环境。五是实施重点污染治理,提升城市环卫保洁水平。六是完善市政设施建设,提升城市绿化水平。七是加强城镇低收入住房保障,实施农村危房改造。八是实施文化惠民,推广旅游文化。九是加大对特殊群体帮扶力度,提高重点优抚补助标准。十是加大公共交通投入,方便市民出行。

(六)全面履行政府职能,着力建设法治服务廉洁政府

建设法治政府。自觉接受市人大及其常委会的法律监督、工作监督和市政协的民主监督,认真办理人大议案建议和政协提案,广泛听取民主党派、工商联、无党派人士和工会、共青团、妇联等人民团体的意见建议。深入推进科学、民主立法。推进城市综合执法体制改革,强化区(县)、镇(街道)执法能力和责任。深入开展“七五普法”,加强司法行政工作。加强公益性法律服务保障,完善一村(社区)一法律顾问工作机制,探索建立一校一法律顾问制度。积极创建国家社会信用体系示范城市,坚持政府率先用信,加强公务员诚信管理。

建设服务政府。深化“一门式一网式”政府服务模式改革,推动全市行政许可、公共服务事项全部进驻实体政务大厅,实现“前台综合受理、后台分类审批、统一窗口出件”。再造行政审批流程,积极推行网上“一表登记、并联审批、联合审批”,完善基层公共服务平台建设。搭建市投资项目在线审批平台,实现投资项目网上办理和信息集中共享,并与国家、省平台数据纵向贯通。深化商事登记制度改革,进一步放宽住所登记条件、推行简易注销、探索证照分离,加快推进全程电子化改革。推动“个转企、企升规、规改股、股上市”,促进市场主体增量提质。依托“汕头政府在线”和网上办事大厅平台,推进企业专属网页、市民个人网页、电子证照库和政务信息资源共享平台建设。进一步完善12345政府服务热线和公共资源交易平台,运用“汕头企业服务”微信公众号和“汕头政企通”手机客户端,打造全方位多样化综合政务服务体系。强化口岸大通关协同工作机制,推进国际贸易“单一窗口”、广澳港区查验“一站式服务”综合办公大楼建设。

建设廉洁政府。认真落实全面从严治党主体责任要求,坚持党风廉政建设、反腐败工作与经济社会发展一起部署、推动和落实。加强对政府重大决策部署贯彻执行情况,尤其是涉及经济社会发展的重点项目、事关群众切身利益的民生工程的检查督促,坚决纠正上有政策、下有对策,有令不行、有禁不止行为,确保政令畅通。持之以恒推进作风建设,严格落实中央“八项规定”,坚决纠正“四风”问题,严厉整肃公职人员办事“吃拿卡要”和行政不作为、乱作为、慢作为问题。强化监察、审计工作,主动接受各界监督。深化政府信息公开工作,增强政府信息公开的针对性、时效性、准确性和权威性,提高政府工作透明度。切实加强队伍建设,进一步规范公务行为,强化教育培训,严格考核监督,完善奖惩机制,打造一支信念坚定、忠于国家、服务人民、恪尽职守、依法办事、公正廉洁的公务员队伍,营造风清气正的政务环境。

各位代表,汕头全面振兴的伟大事业已经启航,美好前景激励着我们,人民群众期待着我们。让我们更加紧密地团结在以习近平同志为核心的党中央周围,在省委、省政府和市委的坚强领导下,团结带领全市人民,凝聚海内外潮人的智慧和力量,实干担当、奋勇争先,为汕头全面振兴发展而努力奋斗!
